# An Antithrombotic Strategy by Targeting Phospholipase D in Human Platelets

**DOI:** 10.3390/jcm7110440

**Published:** 2018-11-14

**Authors:** Wan Jung Lu, Chi Li Chung, Ray Jade Chen, Li Ting Huang, Li Ming Lien, Chao Chien Chang, Kuan Hung Lin, Joen Rong Sheu

**Affiliations:** 1 Department of Medical Research, Taipei Medical University Hospital, Taipei 110, Taiwan; luwj@tmu.edu.tw; 2 Department of Pharmacology, School of Medicine, College of Medicine, Taipei Medical University, Taipei 110, Taiwan; tiffany4441@gmail.com (L.T.H.); change@seed.net.tw (C.C.C.); 3 Graduate Institute of Metabolism and Obesity Sciences, College of Public Health and Nutrition, Taipei Medical University, Taipei 110, Taiwan; 4 Division of Pulmonary Medicine, Department of Internal Medicine, Taipei Medical University Hospital, Taipei 110, Taiwan; clchung@tmu.edu.tw; 5 School of Respiratory Therapy, College of Medicine, Taipei Medical University, Taipei 110, Taiwan; 6 Division of General Surgery, Department of Surgery, Taipei Medical University Hospital, Taipei 110, Taiwan; rayjchen@tmu.edu.tw; 7 School of Medicine, College of Medicine, Taipei Medical University, Taipei 110, Taiwan; m002177@ms.skh.org.tw; 8 Department of Neurology, Shin-Kong Wu Ho-Su Memorial Hospital, Taipei 111, Taiwan; 9 Central Laboratory, Shin-Kong Wu Ho-Su Memorial Hospital, Taipei 111, Taiwan; 10 Institute of Biomedical Sciences, Mackay Medical College, New Taipei City 252, Taiwan; 11 Graduate Institute of Medical Sciences, College of Medicine, Taipei Medical University, Taipei 110, Taiwan

**Keywords:** phospholipase D, platelet activation, clot retraction, thrombus formation

## Abstract

Phospholipase D (PLD) is involved in many biological processes. PLD1 plays a crucial role in regulating the platelet activity of mice; however, the role of PLD in the platelet activation of humans remains unclear. Therefore, we investigated whether PLD is involved in the platelet activation of humans. Our data revealed that inhibition of PLD1 or PLD2 using pharmacological inhibitors effectively inhibits platelet aggregation in humans. However, previous studies have showed that PLD1 or PLD2 deletion did not affect mouse platelet aggregation *in vitro*, whereas only PLD1 deletion inhibited thrombus formation *in vivo*. Intriguingly, our data also showed that the pharmacological inhibition of PLD1 or PLD2 does not affect mouse platelet aggregation *in vitro*, whereas the inhibition of only PLD1 delayed thrombus formation *in vivo*. These findings indicate that PLD may play differential roles in humans and mice. In humans, PLD inhibition attenuates platelet activation, adhesion, spreading, and clot retraction. For the first time, we demonstrated that PLD1 and PLD2 are essential for platelet activation in humans, and PLD plays different roles in platelet function in humans and mice. Our findings also indicate that targeting PLD may provide a safe and alternative therapeutic approach for preventing thromboembolic disorders.

## 1. Introduction

Platelets play a crucial role in normal haemostasis. When blood vessels are damaged, platelets are activated and form a platelet plug at the injury site to prevent blood loss. By contrast, they can also cause vessel occlusion under pathological conditions or with diseases such as inflammation and diabetes mellitus. To date, clinical antiplatelet drugs, such as aspirin and clopidogrel, have been commonly used to prevent secondary stroke but account for only a 20% reduction of all recurrent strokes [1]. For improved safety and efficacy, scientists have exerted considerable efforts to develop new antiplatelet drugs. For example, in 2014, the US Food and Drug Administration approved the thrombin receptor (protease-activated receptor 1) antagonist vorapaxar, a new category of antiplatelet drugs, for preventing secondary stroke. These novel antiplatelet drugs offer alternative therapeutic strategies for reducing recurrent stroke, depending on their mechanisms of antiplatelet activity.

Phospholipase D (PLD) is an enzyme that hydrolyzes phosphatidylcholine to generate choline and the second messenger signaling lipid phosphatidic acid (PA) in response to various stimuli, such as growth factors and neurotransmitters [2]. PLD has several isoforms, and PLD1 and PLD2 were proposed to be involved in many biological processes and diseases including cancer, immunity, and Alzheimer’s disease [2]. In human platelets, PLD mainly exhibits two isoforms, PLD1 and PLD2, which translocate to the plasma membrane during platelet activation and produce PA [3]. Moreover, PLD activity reportedly increases on stimulation with collagen and thrombin [3]. Several studies have investigated the role of PLD1 and PLD2 in platelet activation and thrombus formation using *Pld1^−/−^* and *Pld2^−/−^* mice [4,5]. Platelets from *Pld1^−/−^* mice reportedly exhibit impaired integrin αIIbβ3 activation and shear stress-induced thrombus formation but do not exhibit impaired granule release or platelet aggregation [4]. Furthermore, Thielmann et al. [5] found that PLD2 deletion did not affect agonist-induced integrin αIIbβ3 activation, granule release, and platelet aggregation. However, genetic deletion or pharmacological inhibition of both PLD1 and PLD2 synergistically blocked integrin αIIbβ3 activation and α-granule release but did not affect platelet aggregation [5,6]. In addition, pharmacological inhibition of PLD protects mice against occlusive thrombus formation and ischaemic stroke in the absence of major intracerebral haemorrhage or prolongation of bleeding time, suggesting that pharmacological PLD inhibition is a safe therapeutic strategy for preventing arterial thrombosis and ischaemic stroke [6].

Although evidence suggests that PLD is involved in thrombus formation in mice, its effects on human platelets remain unclear. In our preliminary results, we found that PLD1 or PLD2 inhibition using the selective pharmacological PLD1 inhibitor VU1, whose official code is VU0155069, and PLD2 inhibitor VU2, whose official code is VU0364739 [7,8], respectively, blocked human platelet aggregation and granule release, suggesting that the role of PLD in the platelet function may be different in humans and mice. Thus, in this study, the function and mechanism of PLD underlying human platelet activation were determined for the first time.

## 2. Materials and Methods

### 2.1. Materials

VU0155069 (VU1) and VU0364739 (VU2) were purchased from Tocris Bioscience (Bristol, UK). Thrombin, collagen, and U46619 were purchased from Chrono-Log (Havertown, PA, USA). The fluorescein isothiocyanate (FITC)-conjugated anti-P-selectin antibody was purchased from Biolegend (San Diego, CA, USA). The anti-phospho-PLD1 (Thr^147^), anti-phospho-p38 mitogen-activated protein kinase (MAPK) (Ser^180^/Tyr^182^), anti-phospho-p44/42 MAPK (extracellular signal-regulated protein kinases 1 and 2 [ERK1/2]) (Thr^202^/Tyr^204^), anti-c-Jun N-terminal kinase (JNK), and anti-phospho-Akt (Ser^473^) polyclonal antibodies (pAbs) and the anti-PLD2, anti-p38 MAPK, anti-p44/42 MAPK, anti-phospho JNK (Thr^183^/Tyr^185^), and anti-Akt monoclonal antibodies (mAbs) were purchased from Cell Signaling (Beverly, MA, USA). The anti-phospho-PLD2 (Tyr^169^) pAb was purchased from Abcam (Cambridge, UK). The anti-PLD1 mAb was purchased from Santa Cruz Biotechnology (Dallas, TX, USA). The Hybond-P polyvinylidene difluoride membrane, an enhanced chemiluminescence (ECL) Western blotting detection reagent and analysis system, horseradish peroxidase (HRP)-conjugated donkey anti-rabbit immunoglobulin G (IgG), and sheep anti-mouse IgG were purchased from Amersham (Buckinghamshire, UK). VU1 and VU2 were dissolved in dimethyl sulphoxide (DMSO) and stored at 4 °C until use.

### 2.2. Platelet Aggregation and Adenosine Triphosphate Release

This study was approved by the Institutional Review Board of Shin Kong Wu Ho-Su Memorial Hospital (Approval No. 20160705R) and conformed to the principles outlined in the Declaration of Helsinki. All volunteers provided informed consent. Human platelet suspensions were prepared as previously described [9,10]. In brief, blood was collected from healthy volunteers, who had taken no medicine during the preceding two weeks, and mixed with an acid–citrate–dextrose solution (9:1, *v*/*v*). After centrifugation, the supernatant (platelet-rich plasma) was supplemented with prostaglandin E1 (0.5 μM) and heparin (6.4 IU/mL). Washed platelets were suspended in Tyrode’s solution containing bovine serum albumin (BSA; 3.5 mg/mL). The final concentration of Ca^2+^ in Tyrode’s solution was 1 mM.

Platelet aggregation was measured through turbidimetry [9,10] using a Lumi-Aggregometer (Payton, Scarborough, ON, Canada), and adenosine triphosphate (ATP) release was detected through the measurement of luminescence using the F-4500 spectrometer (Hitachi, Osaka, Japan). Platelet suspensions (3.6 × 10^8^ cells/mL) were preincubated with various concentrations of VU1, VU2, or an isovolumetric solvent control (0.1% DMSO, final concentration) for 3 min before the addition of agonists. The reaction was allowed to proceed for 6 min.

### 2.3. Flow Cytometry

The flow cytometry experiment was performed as described previously [11]. In brief, the platelet suspensions (1 × 10^6^ platelets/mL) were preincubated with VU1, VU2, or 0.1% DMSO for 3 min, and subsequently, thrombin (0.01 U/mL) was added for 6 min in glass cuvettes at 37 °C. After the reactions, the platelet suspensions were stained with P-selectin–FITC for 30 min. A final volume of 1 mL was used for an immediate analysis through flow cytometry (Becton Dickinson, FACScan Syst., San Jose, CA, USA). Data were collected from 10,000 platelets per experimental group. All experiments were repeated at least three times to ensure reproducibility.

### 2.4. Immunoblotting Study

Washed platelets (1.2 × 10^9^ cells/mL) were preincubated with VU1, VU2, or 0.1% DMSO for 3 min, and thrombin (0.01 U/mL) was then added to trigger platelet activation. The reaction was stopped, and the platelets were immediately re-suspended in 200 μL of lysis buffer for 1 h. Lysates were centrifuged at 5000× *g* for 5 min. Samples containing 80 μg of protein were separated using 12% sodium dodecylsulphate polyacrylamide gel electrophoresis; proteins were electrotransferred using semidry transfer (Bio-Rad, Hercules, CA, USA). Blots were blocked with Tris-buffered saline, 0.1% Tween 20 (TBST; 10 mM Tris-base, 100 mM NaCl, and 0.01% Tween 20) containing 5% BSA for 1 h and then probed with various primary antibodies. Membranes were incubated with HRP-conjugated anti-mouse IgG or anti-rabbit IgG (diluted 1:3000 in TBST) for 1 h. Immunoreactive bands were detected using an ECL system. The bar graph depicts the ratios of semi-quantitative results obtained through scanning of reactive bands and quantifying the optical density using videodensitometry (Bio-Profil; Biolight Windows Application V2000.01, Vilber Lourmat, Marne La Vallée, France).

### 2.5. Platelet Adhesion and Spreading on Immobilized Fibrinogen

This experiment was performed as previously described with minor modification [12]. In brief, platelet suspensions (3 × 10^7^ cells/mL) were preincubated with VU1, VU2, or 0.1% DMSO for 3 min at 37 °C, then transferred to fibrinogen (100 μg/mL)-coated slides, and allowed to adhere and spread for 1.5 h at 37 °C. The slides were immediately washed to detach non-adherent platelets, and adherent platelets were fixed with 4% paraformaldehyde. Samples were blocked with 5% BSA in PBS and incubated with FITC-phalloidin for 1 h at 37 °C. Fluorescence images were obtained using a Leica TCS SP5 confocal spectral microscope (Leica Microsystems, Wetzlar, Germany), and the platelet number and size were quantified in three random fields using ImageJ.

### 2.6. Clot Retraction

Platelet suspensions (3.6 × 10^8^ cells/mL) were pretreated with VU1, VU2, or 0.1% DMSO for 3 min, and subsequently, clot retraction was initiated with thrombin (0.01 U/mL) in the presence of fibrinogen (2 mg/mL) and CaCl_2_ (1 mM). Clot retraction was allowed to proceed at 37 °C in an aggregometer tube and photographed at the indicated times.

### 2.7. Animals

ICR and C57BL/6 mice (20–25 g, male, 5–6 weeks old) were obtained from BioLasco (Taipei, Taiwan). All procedures were approved through the Affidavit of Approval of Animal Use Protocol of Taipei Medical University (Approval No. LAC-2016-0195) and were in accordance with the Guide for the Care and Use of Laboratory Animals (Eighth Edition, 2011). The concentration of 5 μM of VU1 and VU2 was chosen and calculated accordingly into mouse doses VU1 (2.7 mg/kg) and VU2 (2.5 mg/kg), respectively [13].

### 2.8. Sodium Fluorescein-Induced Platelet Thrombus Formation in Mesenteric Microvessels of Mice

Thrombus formation was assessed as previously described [11]. Mice were anaesthetized using a mixture containing 75% air and 3% isoflurane maintained in 25% oxygen, and the external jugular vein was cannulated with a polyethylene-10 tube for intravenously administering dyes and drugs. Venules (30–40 mm) were selected for irradiation at wavelengths of <520 nm to produce a microthrombus. Either VU1 (2.7 mg/kg) or VU2 (2.5 mg/kg) was administered for 10 min before the administration of sodium fluorescein (20 mg/kg), and the time required to occlude the microvessel through thrombus formation (occlusion time) was recorded. The dose for mice was accordingly converted from the dose for humans [13].

### 2.9. Tail Bleeding Time

Mice were anaesthetized with a mixture containing 75% air and 3% isoflurane maintained in 25% oxygen. They were intraperitoneally administrated with saline (control), DMSO (solvent control), VU1 (2.7 mg/kg) or VU2 (2.5 mg/kg) for 30 min. Immediately, bleeding was induced by severing the tail 3 mm from the tail tip, and the bleeding tail stump was immersed in saline. The bleeding time was continually recorded until no sign of bleeding was observed for at least 10 s. The dose for mice was accordingly converted from the dose for humans [13].

### 2.10. Data Analysis

The experimental results are expressed as means ± standard error of the mean (SEM), and are accompanied by the number of observations (*n*). Values of n refer to the number of experiments, each of which was conducted using different blood donors. All experimental results were assessed through analysis of variance (ANOVA). If ANOVA indicated significant differences in the group means, each group was compared using the Newman–Keuls method. Survival curves were plotted using the Kaplan–Meier method, and the groups were compared using the log-rank test. A value of *p* < 0.05 was considered statistically significant.

## 3. Results

### 3.1. PLD Plays Differential Roles in Platelet Functions in Humans and Mice

**Figure 1:**
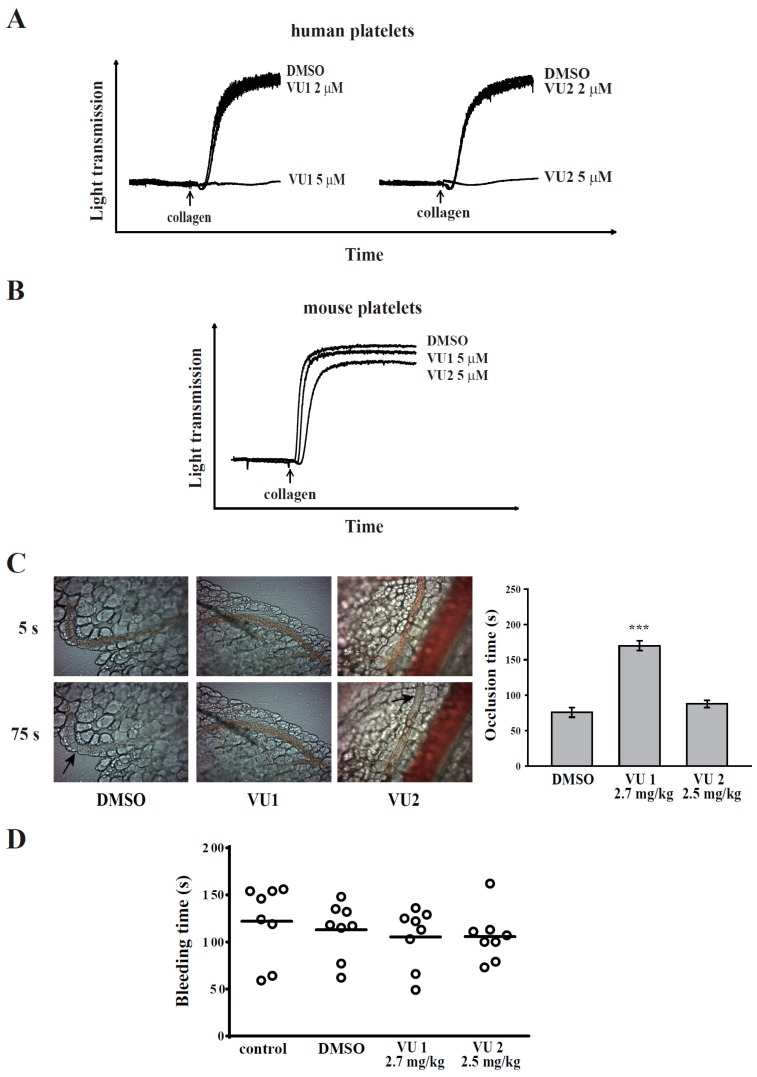
Effects of phospholipase D (PLD)1 and PLD2 on platelet aggregation and thrombus formation. Washed (**A**) human platelets (3.6 × 10^8^ cells/mL) and (**B**) mouse platelets (1 × 10^8^ cells/mL) were preincubated with DMSO (solvent control), the PLD1 inhibitor VU1 (2 or 5 μM), or the PLD2 inhibitor VU2 (2 or 5 μM) and then stimulated using collagen (1 μg/mL) to trigger platelet aggregation. (**C**) Mice received an intravenous bolus of DMSO, VU1 (2.7 mg/kg), or VU2 (2.5 mg/kg) for 30 min before the administration of sodium fluorescein; subsequently, mesenteric venules were irradiated to induce microthrombus formation. Arrows indicate thrombus. (**D**) Bleeding was induced by severing the tail at 3 mm from the tail tip, and the bleeding tail stump was immersed in saline. The bleeding time was continually recorded until no sign of bleeding was observed for at least 10 s. Each point in the scatter plot graph represents a mouse. The profiles (**A**,**B**) are representative examples of three similar experiments. Data (**C**,**D**) are presented as the mean ± standard error of the mean (SEM) (**C**, *n* = 6; **D**, *n* = 8). *** *p* < 0.001, compared with the DMSO group.

Previously, the selective PLD1 inhibitor VU1 and PLD2 inhibitor VU2 have been used to evaluate the roles of PLD1 and PLD2, respectively, in a variety of cells [7,8,14]. Moreover, a concentration of 5–10 μM of both inhibitors was most commonly used. Therefore, we first investigated whether both inhibitors at 5 μM could affect human platelet aggregation. As shown in Figure 1A, at a concentration of 5 μM, VU1 or VU2 completely inhibited collagen-induced platelet aggregation. However, PLD knockout mice [4,5] showed that PLD deletion did not affect mouse platelet aggregation, whereas the deletion of only PLD1 (not PLD2) exhibited protective effects in thrombus formation. Here, the effect of both inhibitors on mouse platelet aggregation was determined. Intriguingly, our data also showed that both inhibitors did not interfere with collagen-induced platelet aggregation (Figure 1B). Moreover, only VU1, but not VU2, could effectively prevent thrombus formation in mice (Figure 1C). In addition, VU1 and VU2 did not affect normal haemostasis (Figure 1D). Thus, PLD may have different functions in humans and mice, but it is certain that both PLD1 and PLD2 are essential for platelet activation in humans.

### 3.2. PLD Inhibition May Reduce ATP Release and P-Selectin Secretion in Thrombin-Induced Human Platelet Activation

**Figure 2:**
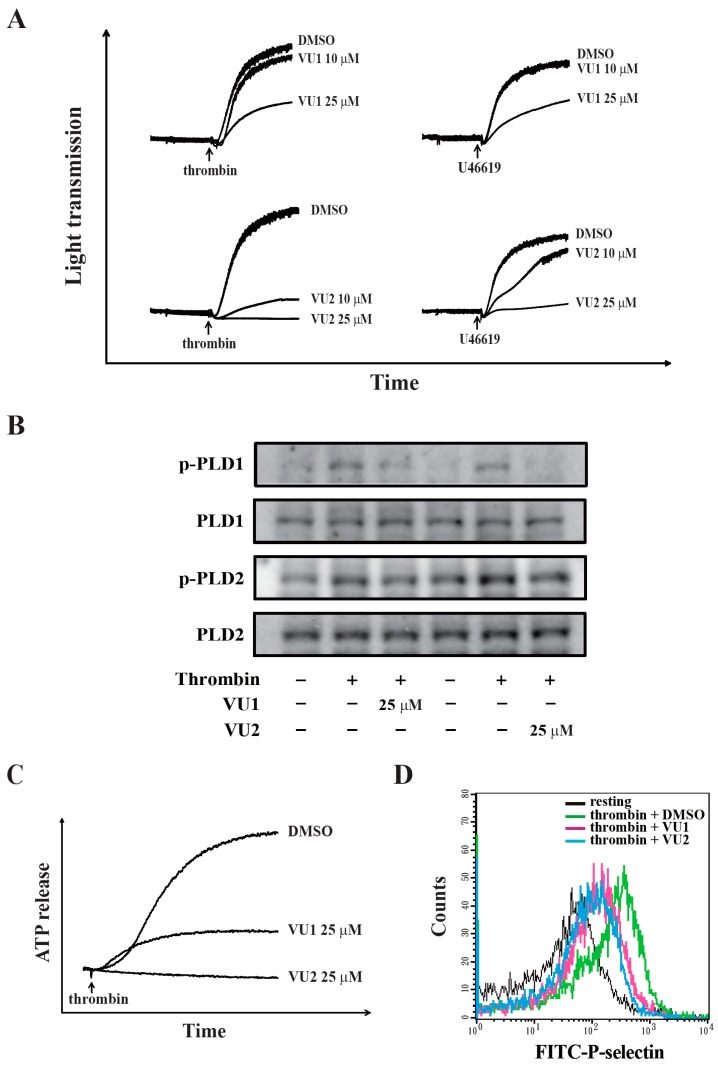
Pharmacological inhibition of PLD mediated the reduction of human platelet aggregation and granule release. (**A**,**B**) Washed human platelets (3.6 × 10^8^ cells/mL) were preincubated with DMSO (solvent control), VU1 (10 and 25 μM), or VU2 (10 and 25 μM) and then stimulated using thrombin (0.01 U/mL) and U46619 (1 μM) to trigger (**A**) platelet aggregation and the (**B**) phosphorylation of PLD1 and 2. Effects of VU1 (25 μM) and VU2 (25 μM) on (**C**) thrombin-induced adenosine triphosphate (ATP) release and (**D**) P-selectin secretion were characterized by the detection of chemiluminescent emission from the luciferin–luciferase reaction and the fluorescence of P-selectin–fluorescein isothiocyanate (FITC)–antibody through flow cytometry, respectively. The profiles (**A**–**D**) are representative examples of three similar experiments.

We found that VU1 and VU2 at a concentration of 25 μM effectively reduced thrombin- and U46619-induced platelet aggregation (Figure 2A). To confirm the selectivity of VU1 and VU2, we further examined the phosphorylation of PLD1 and PLD2 at Thr^147^ and Tyr^169^, respectively; these sites have been reported to be associated with their activities [15,16]. We found that VU1 and VU2 dose-dependently (10–25 μM) inhibited the phosphorylation of PLD1 and PLD2, respectively (Figure S1A). Moreover, VU1 and VU2 at 25 μM markedly inhibited the phosphorylation of PLD1 and PLD2 (Figure 2B), indicating that VU1 (25 μM) and VU2 (25 μM) non-selectively inhibited both PLD1 and PLD2. These findings suggest that the concurrent inhibition of PLD1 and PLD2 may exert an optimal effect inhibiting all agonist-induced platelet aggregation. Therefore, in this study, we focused on the role of PLD, not specific to PLD1 or PLD2, in human platelet activation. The concentration of 25 μM of VU1 and VU2 was accordingly used to determine the role of PLD in human platelets in the following experiments. As shown in Figure 2C,D, VU1 (25 μM) and VU2 (25 μM) reduced thrombin-induced ATP release and P-selectin secretion, suggesting that PLD regulates granule release. Our data also support the fact that both PLDs are involved in platelet activation, granule release, and aggregation in human platelets. In addition, VU1 and VU2 (10–25 μM) did not exhibit cytotoxic effects on human platelets, as detected by the lactate dehydrogenase (LDH) assay (Figure S2), indicating that the VU1- and VU2-mediated inhibition of platelet activation is not due to the cytotoxicity.

### 3.3. PLD May Regulate Akt and MAPK Activation in Thrombin-Induced Platelet Activation

**Figure 3:**
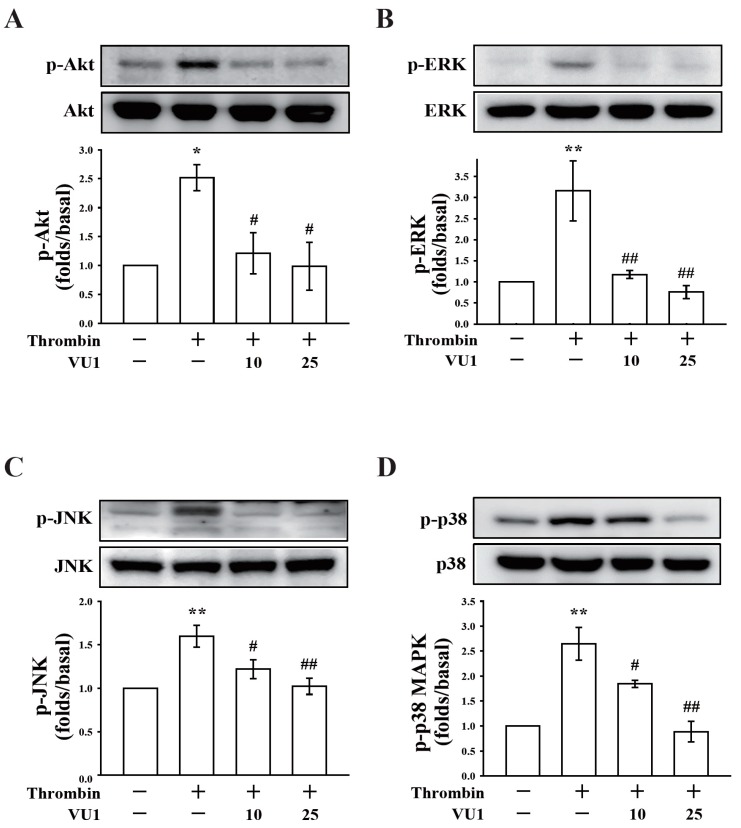
Effects of VU1 on the phosphorylation of Akt and mitogen-activated protein kinases (MAPKs). Washed platelets (1.2 × 10^9^ cells/mL) were preincubated with DMSO or VU1 (10 and 25 μM), and thrombin (0.01 U/mL) was subsequently added to trigger the phosphorylation of Akt, extracellular signal-regulated protein kinase (ERK), anti-c-Jun N-terminal kinase (JNK), and p38 MAPK. Cells were then collected, and subcellular extracts were analyzed through Western blotting. Data (**A**–**D**) are presented as means ± SEM (*n* = 3). * *p* < 0.05 and ** *p* < 0.01, compared with the resting group; # *p* < 0.05 and ## *p* < 0.01, compared with the thrombin (positive) group.

**Figure 4:**
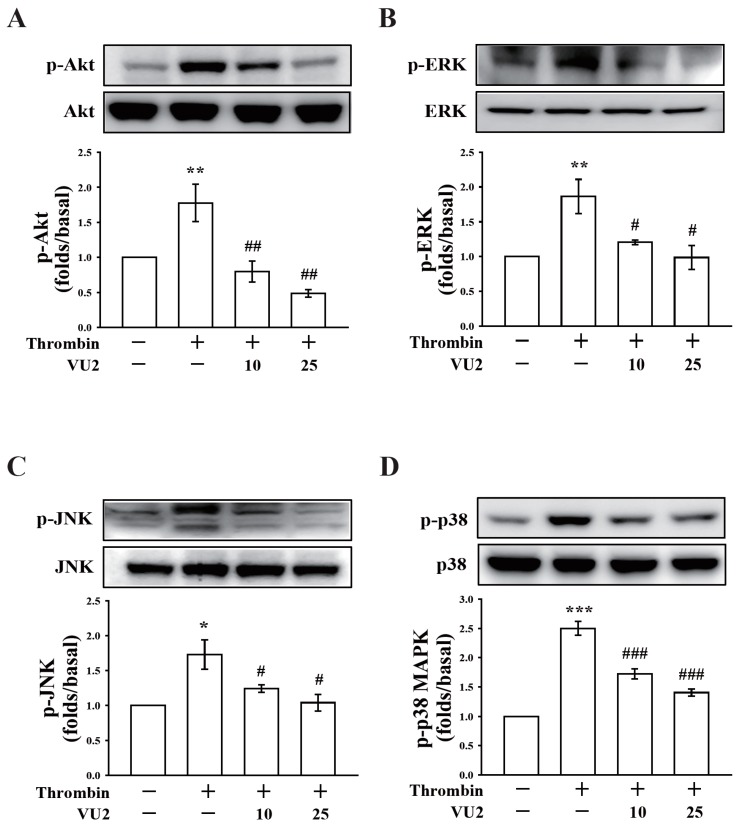
Effects of VU2 on the phosphorylation of Akt and MAPKs. Washed platelets (1.2 × 10^9^ cells/mL) were preincubated with DMSO or VU2 (10 and 25 μM), and thrombin (0.01 U/mL) was subsequently added to trigger the phosphorylation of Akt, ERK, JNK, and p38 MAPK. Cells were then collected, and subcellular extracts were analyzed through Western blotting. Data (**A**–**D**) are presented as means ± SEM (*n* = 3). * *p* < 0.05, ** *p* < 0.01, and *** *p* < 0.001, compared with the resting group; # *p* < 0.05, ## *p* < 0.01, and ### *p* < 0.001, compared with the thrombin (positive) group.

In this study, we determined whether PLD is involved in Akt signaling, which reportedly contributes to platelet activation and supports thrombus formation [17]. As shown in Figure 3A and Figure 4A, thrombin markedly stimulated Akt phosphorylation, which was reversed using VU1 (25 μM) and VU2 (25 μM), suggesting that PLD regulates platelet activation through Akt signaling in humans.

We also determined the role of PLD in the activation of MAPKs, including ERK, JNK, and p38 MAPK, which play key roles in stimulating the secretion of platelet granules [12]. Our results show that thrombin markedly stimulated the phosphorylation of ERK, JNK, and p38 MAPK, which was reversed using VU1 (25 μM) and VU2 (25 μM) (Figure 3B–D; Figure 4B–D). Thus, PLD regulates granule secretion through the MAPK pathway in activated human platelets.

### 3.4. PLD May Maintain Platelet Adhesion, Spreading, and Clot Retraction through Outside-In Signaling

**Figure 5:**
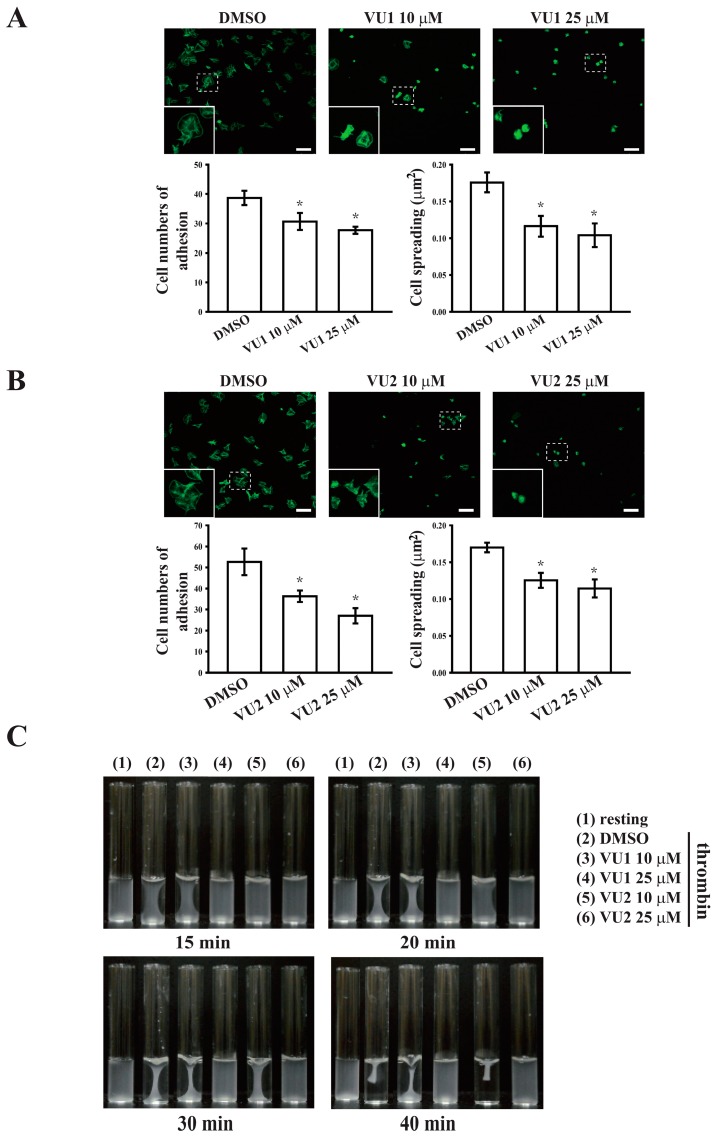
Regulatory effects of PLD on platelet adhesion, spreading, and clot retraction. (**A**,**B**) Washed platelets (3 × 10^7^ cells/mL) were preincubated with DMSO, VU1 (10 and 25 μM), or VU2 (10 and 25 μM) and allowed to adhere and spread on immobilized fibrinogen for 1.5 h. After fixation, cells were stained with FITC-phalloidin, and images were obtained using a confocal microscope. Scale bar: 10 μm. The inset indicates the high magnification image of the region marked by dashed box. (**C**) Platelet suspensions (3.6 × 10^8^ cells/mL) were pretreated with DMSO, VU1 (10 and 25 μM), or VU2 (10 and 25 μM) for 3 min, and subsequently, clot retraction was initiated using thrombin (0.01 U/mL) in the presence of fibrinogen and CaCl_2_. Clot retraction was allowed to proceed at 37 °C in an aggregometer tube and photographed at the indicated times. Data (**A**,**B**) are presented as means ± SEM (*n* = 3). * *p* < 0.05, compared with the DMSO (solvent control) group. Profiles (**C**) are representative examples of three similar experiments.

Platelet adhesion, spreading, and clot retraction generally require ligand binding to integrin αIIbβ3, and this binding mediates outside-in signaling, which is crucial in thrombosis [18,19]. Therefore, these functional tests were also performed. As shown in Figure 5A,B, platelets adhered and spread effectively on immobilized fibrinogen, whereas VU1 and VU2 significantly reduced platelet adhesion and spreading (*p* < 0.05). In addition, the platelet suspension initiated clot retraction 15 min after thrombin addition, and full clot retraction was observed at 40 min after addition (Figure 5C). Moreover, VU1 (25 μM) and VU2 (25 μM) completely prevented clot retraction within 40 min of the reaction. These findings indicate that PLD inhibition may interfere with platelet adhesion, spreading, and clot retraction and ultimately impair thrombus formation.

## 4. Discussion

**Figure 6:**
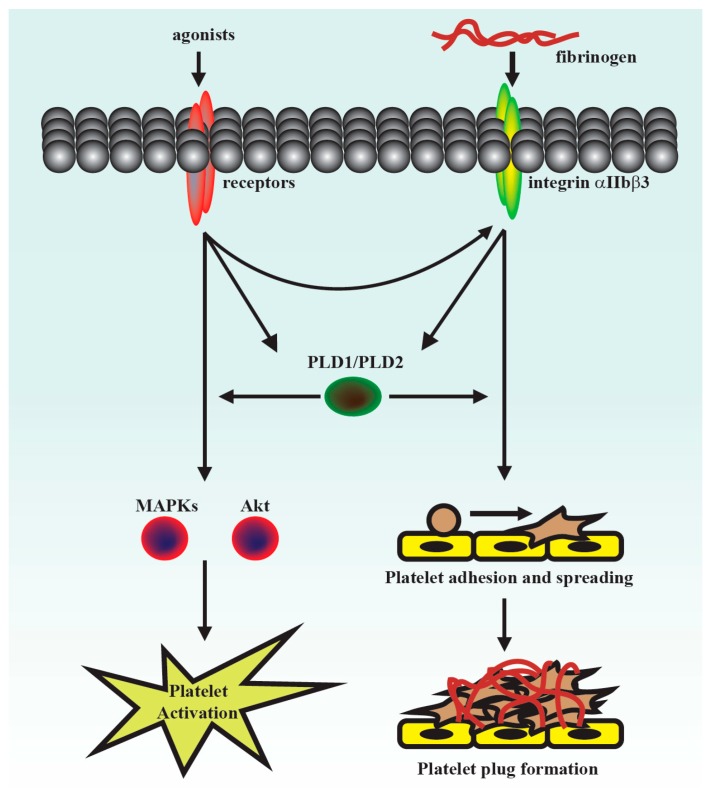
Hypothetical scheme of PLD (PLD1 and PLD2) involvement in human platelet activation. PLD may be involved in the activation of Akt and MAPK, leading to granule release and subsequent inside-out signaling-mediated integrin αIIbβ3 activation. It may also be involved in outside-in signaling of integrin αIIbβ3 and support platelet adhesion, spreading, and plug formation, thus stabilizing thrombus formation.

This was the first study to demonstrate that PLD maintains platelet activation, adhesion, spreading, and clot retraction in humans (Figure 6). Moreover, our data also suggested that PLD has different effects on platelet activation in humans and mice. We found that PLD1 and PLD2 are essential for platelet activation in humans, and PLD1 is more crucial for platelet activation in mice.

PLD is involved in a wide range of cell biological processes, including degranulation, endocytosis, cell invasion, and cytoskeletal reorganization [20]. Furthermore, it is involved in many diseases, such as cancer and Alzheimer’s disease. For example, PLD regulates tumourigenesis and cancer cell survival and invasion [7,21,22]. In addition, PLD1, but not PLD2, regulates ischaemic cardiovascular events in mice [4,5,6]. However, the role of PLD in human platelets remains unclear. Thus, this study further clarified the role of PLD in human platelet activation.

Previous studies have reported that PLD1 deletion slightly lowers integrin αIIbβ3 activation but does not inhibit agonist-induced granule release or platelet aggregation in mice [4,5]. Moreover, the absence of PLD2 does not alter platelet function [5]. In addition, deletion of both PLD1 and PLD2 partially blocks granule release but does not inhibit platelet aggregation in mice [4,5]. However, our data showed that PLD1 and PLD2 inhibitors at a concentration of 5 μM markedly inhibited human platelet aggregation, but did not affect mouse platelet aggregation. In addition, only the PLD1 inhibitor (2.7 mg/kg), but not the PLD2 inhibitor (2.5 mg/kg), significantly delayed thrombus formation that is consistent with previous studies that have shown that the genetic deletion of only PLD1, but not PLD2, impairs thrombus formation [4,5]. These findings suggest that PLD has differential roles in the platelet function of humans and mice. Either PLD1 or PLD2 play a crucial role in human platelet activation; PLD1 plays a more vital role in platelet activation and thrombus formation in mice. In fact, the discrepancy of platelet activation in response to adenosine diphosphate (ADP) and thrombin was previously reported between humans and mice [23], indicating that the processes of platelet activation may differ in different species. Moreover, Mestas and Hughes have reported that immune responses are different in humans and mice [24]. They found that delayed-type hypersensitivity response tends to be more neutrophil rich in humans than in mice [24]. They also suggested that these differences should be taken into account when using mice as preclinical models of human disease [24]. Thus, it is crucial to understand the differences between humans and mice.

The selective PLD1 inhibitor VU0155069 (VU1) and PLD2 inhibitor VU0364739 (VU2) have been used to evaluate the roles of PLD1 and PLD2, respectively, in a variety of cells [7,8,14], in which the concentrations (5–10 μM) of both inhibitors were used. In the present study, the concentrations of 10 and 25 μM of VU1 and VU2 were used, and the data showed that VU1 and VU2 at the concentration of 25 μM more effectively prevented platelet aggregation-induced by collagen, thrombin, and U46619. Interestingly, we found that VU1 (10 μM) could inhibit PLD1, but not PLD2, phosphorylation and that VU2 (10 μM) could inhibit PLD2, but not PLD1, phosphorylation (Figure S1), indicating that VU1 and VU2 below the concentration of 10 μM exerted a more selective inhibition of PLD1 and PLD2, respectively, in human platelets. However, VU1 and VU2 at the concentration of 25 μM non-selectively inhibited PLD2 and PLD1 activity, respectively, in human platelets. These findings suggest that concurrent inhibition of PLD1 and PLD2 may achieve an optimal antiplatelet effect. Moreover, we also excluded this possibility that the VU1- or VU2-mediated inhibition of PLD phosphorylation is due to protein kinase C (PKC) inhibition (Figure S3). Thus, the concentration of 25 μM of VU1 and VU2 used in the present study is aimed to elucidate that the concurrent inhibition of PLD1 and PLD2 may be a therapeutic strategy for preventing human platelet activation. Additionally, 4-Fluoro-N-(2-(4-(5-fluoro-1H-indol-1-yl) piperidin-1-yl) ethyl) benzamide, 5-Fluoro-2-indolyl des-chlorohalopemide (FIPI), a PLD1/PLD2 inhibitor, has been used in platelet research in mice [6]. However, this inhibitor has been reported to enhance thrombin-induced human platelet aggregation [25]. Here, we also showed that FIPI could enhance collagen-induced human platelet aggregation (Figure S4). These findings indicate that FIPI may not be a suitable compound to investigate the role of PLD in human platelets.

Our present study revealed that PLD (PLD1 and PLD2) could regulate human platelet activation through inside-out signaling. Our data showed that PLD inhibition prevented platelet aggregation induced through agonists, such as collagen, thrombin, and the thromboxane A_2_ analogue U46619, suggesting that PLD regulates receptor-mediated downstream signaling. Moreover, PLD inhibition blocks granule release. To determine the possible mechanisms through which PLD regulates platelet activation, the role of PLD in two signaling pathways, namely the MAPK and Akt pathways, was analyzed in this study. We found that PLD inhibition significantly inhibited the activation of MAPKs and Akt. MAPKs are a family of serine/threonine protein kinases comprising three major subgroups: ERKs, p38 MAPK, and JNKs. Among the MAPKs, ERK2, p38 MAPK, and JNK1 are present in platelets, activated by various agonists, and involved in platelet granule release, aggregation, adhesion, and thrombus formation [26]. In addition, previous studies on *Akt1^−/−^*, *Akt2^−/−^*, and *Akt3^−/−^* mice have indicated that Akt signaling plays a key role in regulating platelet aggregation, granule secretion, and fibrinogen binding [17,27]. Evidence indicates that PLD inhibition can inhibit platelet activation, granule release, and subsequent platelet aggregation, at least in part, through the MAPK and Akt pathways in human platelets.

Platelet activation is involved in several initiation steps. Various platelet agonists (e.g., thrombin and collagen) induce inside-out signaling and trigger ligand binding to integrin αIIbβ3; this binding subsequently activates outside-in signaling, which is crucial for clot retraction and thrombus consolidation [12,18,28]. Outside-in signaling can considerably amplify the platelet response and support stable platelet adhesion, spreading, and clot retraction, ultimately contributing to thrombus stability [12,18,28,29]. This study revealed that PLD inhibition significantly inhibits clot retraction, indicating that PLD may be involved in outside-in signaling. This finding was further confirmed through an assay of platelet adhesion and spreading, which has been used as a measure of outside-in signaling [30]. This assay revealed that PLD inhibition also significantly inhibited platelet adhesion and spreading. Thus, PLD is involved in clot retraction and thrombus consolidation through outside-in signaling. In addition, our study also demonstrated that PLD inhibition did not prolong the tail bleeding. This finding is consistent with previous studies that reported that genetic deletion or pharmacological inhibition of PLD has not caused uncontrolled bleeding [4,5,6]. Thus, a safe therapeutic strategy for preventing arterial thrombosis and ischaemic stroke may be achieved by targeting PLD. On the other hand, the pharmacologic inhibitors of PLD1 and PLD2 were mainly used to define the role of PLD in human platelets. Here, we do not exclude the possibility of nonspecific effects of these inhibitors. However, PLD inhibitors used in this study have been reported to exhibit more potent and selective inhibitory activity in its individual PLD isoforms [7,8]. Moreover, our data showed that VU1 and VU2 at the commonly used concentration of 5 μM markedly inhibited collagen-induced platelet aggregation in humans but not in mice. This finding strongly suggests that PLD may play a different role on platelet activation in humans and mice, though we cannot exclude possible off-target effects of PLD inhibitors. Further investigation is needed to clarify these differences in future work.

## 5. Conclusions

In conclusion, this study demonstrated that both PLD1 and PLD2 are essential for platelet activation in humans, and PLD plays differential roles in platelet function in humans and mice. Furthermore, a safe and alternative therapeutic approach to preventing thromboembolic disorders, such as secondary stroke, may be achieved by targeting PLD.

## Supplementary Materials

The following are available online at http://www.mdpi.com/2077-0383/7/11/440/s1: Figure S1: Effects of VU1 and VU2 on PLD activity. Washed human platelets (1.2 × 10^9^ cells/mL) were preincubated with DMSO (solvent control), VU1 (10 and 25 μM), or VU2 (10 and 25 μM), and thrombin (0.01 U/mL) was subsequently added to trigger the phosphorylation of PLD1 and PLD2. Cells were then collected, and subcellular extracts were analyzed through Western blotting, Figure S2: Effect of VU1 and VU2 on the cytotoxicity of platelets. The platelets (3.6 × 10^8^ cells/mL) were preincubated with Tyrode’s solution (resting), DMSO (solvent control), or various concentrations of VU1 or VU2(10–25 μM) for 10 min at 37 °C, and the supernatant was collected to measure LDH release by the LDH assay kit. LDH activity was expressed as the % of total enzyme activity, which was measured in platelets lysed with 0.5% Triton X-100 (positive control). Profiles are representative of three similar experiments, Figure S3: Effects of VU1 and VU2 on PKC activity. Washed human platelets (1.2 × 10^9^ cells/mL) were preincubated with DMSO (solvent control), VU1 (25 μM), or VU2 (25 μM), and thrombin (0.01 U/mL) was subsequently added to trigger the phosphorylation of PKC substrate. Cells were then collected, and subcellular extracts were analyzed through Western blotting, Figure S4: Effects of FIPI on collagen-mediated platelet aggregation. Washed human platelets (3.6 × 10^8^ cells/mL) were preincubated with DMSO (solvent control) or FIPI (10 μM), and collagen (1 μg/mL) was then added to trigger platelet aggregation.
