# Supplementary material for: An Antithrombotic Strategy by Targeting Phospholipase D in Human Platelets
Source: J Clin Med. 2018 Nov 14;7(11):440. doi: 10.3390/jcm7110440 (PMC6262437; doi:10.3390/jcm7110440)
Supplement: Supplementary file 1 [file jcm-07-00440-s001.pdf]

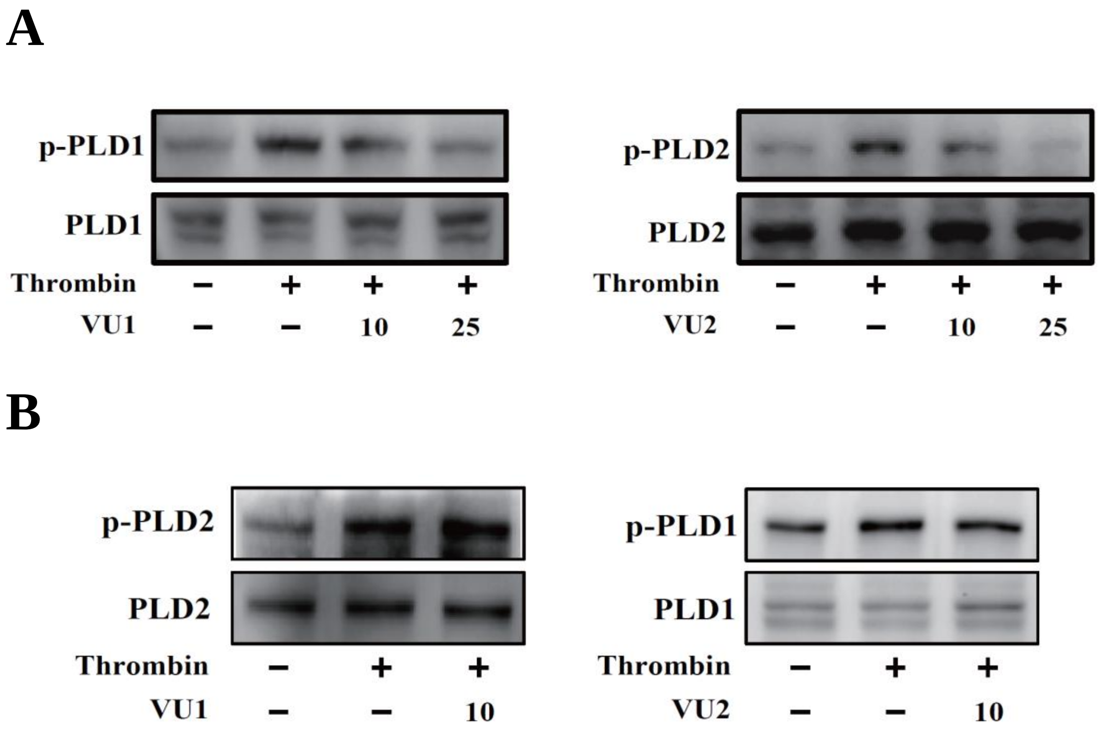

Supplemental Figure 1. Effects of VU1 and VU2 on PLD activity. Washed human platelets (1.2×10^9 cells/mL) were preincubated with DMSO (solvent control), VU1 (10 and 25 μM), or VU2 (10 and 25 μM), and thrombin (0.01 U/mL) was subsequently added to trigger the phosphorylation of PLD1 and PLD2. Cells were then collected, and subcellular extracts were analysed through Western blotting.

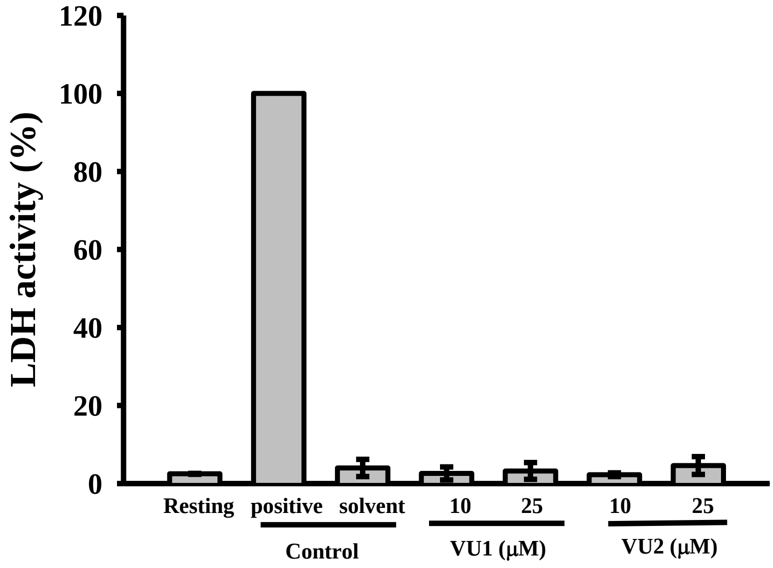

Supplemental Figure 2. Effect of VU1 and VU2 on the cytotoxicity of platelets. The platelets (3.6×10^8 cells/mL) were preincubated with Tyrode's solution (Resting), DMSO (solvent control) or various concentrations of VU1 or VU2 (10-25 μM) for 10 min at 37℃, and the supernatant was collected to measure LDH release by the LDH assay kit. LDH activity was expressed as the % of total enzyme activity, which was measured in platelets lysed with 0.5% Triton X-100 (positive control). Profiles are representative of 3 similar experiments.

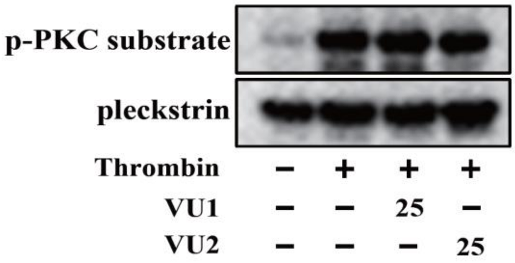

Supplemental Figure 3. Effects of VU1 and VU2 on PKC activity. Washed human platelets (1.2×10^9 cells/mL) were preincubated with DMSO (solvent control), VU1 (25 μM), or VU2 (25 μM), and thrombin (0.01 U/mL) was subsequently added to trigger the phosphorylation of PKC substrate. Cells were then collected, and subcellular extracts were analysed through Western blotting.

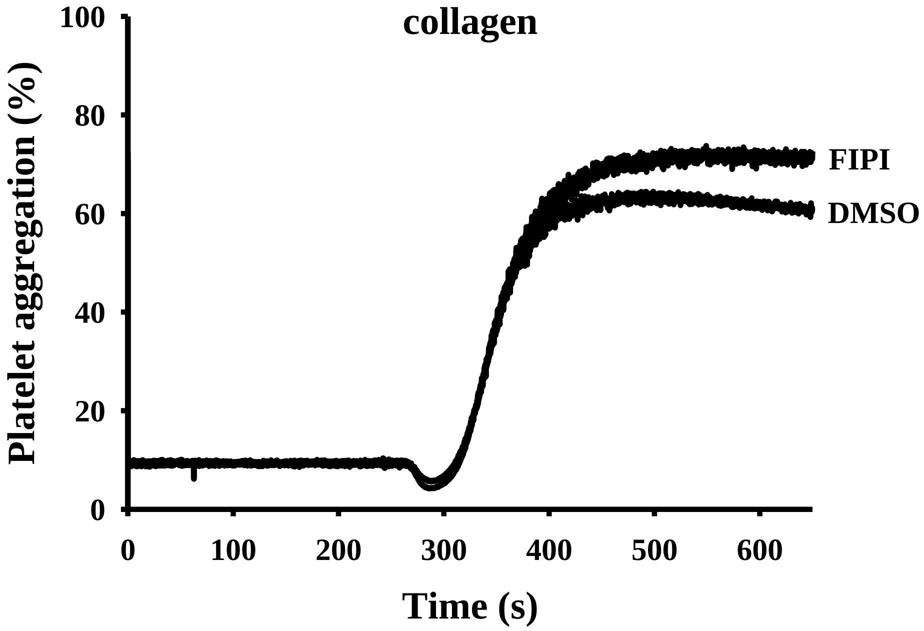

Supplemental Figure 4. Effects of FIPI on collagen-mediated platelet aggregation. Washed human platelets (3.6×10^8 cells/mL) were preincubated with DMSO (solvent control) or FIPI (10 μM), and collagen (1 μg/mL) was then added to trigger platelet aggregation.
